# Correlation of GDFT combined with rehabilitation therapy in DNA damage repair of esophageal cancer cells

**DOI:** 10.3389/fgene.2023.1134994

**Published:** 2023-02-23

**Authors:** Lihua Yan, Yajun Ji

**Affiliations:** ^1^ Rehabilitation Physiotherapy Department, Central Hospital, Cangzhou, Hebei, China; ^2^ No. 1 Anesthesia Department, Central Hospital, Cangzhou, Hebei, China

**Keywords:** esophageal cancer, cellular DNA damage repair, goal directed fluid therapy, combined rehabilitation therapy, EEG signal optimization algorithm

## Abstract

Esophageal cancer is a common malignant tumor with a high incidence and a serious threat to human health. The treatment of esophageal cancer is a complex process, which requires the comprehensive use of a variety of treatment methods. At present, the treatment of esophageal cancer mainly includes surgery, radiotherapy, chemotherapy and immunotherapy. The research on the treatment of cancer cells based on Goal directed fluid therapy (GDFT) combined with rehabilitation therapy is the focus of the current society. This paper proposed a study on DNA damage repair of cancer cells based on goal directed fluid therapy combined with rehabilitation therapy, aiming to optimize the traditional treatment of esophageal cancer by using goal directed fluid therapy technology. The algorithm proposed in this paper was an electroencephalogram (EEG) signal optimization algorithm based on combined rehabilitation therapy. Through this algorithm, the electroencephalogram signal could be optimized. The algorithm could speed up signal processing, and improve signal reliability and stability by reducing the influence of interference signals and improving the signal to noise ratio. These optimization measures could better help researchers analyze and understand electroencephalogram signals, so as to help better study brain functions and diseases. Through the test and investigation on the treatment of cancer cells based on goal directed fluid therapy combined with rehabilitation therapy, the results showed that the blood transfusion volume of goal directed fluid therapy treatment and conventional treatment was 251.5 mL and 288.3 mL respectively. This showed that after goal directed fluid therapy treatment, the input amount of various medical fluids was relatively reduced, and the use of medical fluids was more economical. In addition, their bleeding volumes were 295.2 mL and 324.4 mL, respectively. Urine volume was 382.3 mL and 418.1 mL respectively. This showed that after goal directed fluid therapy treatment, the patient’s blood loss and urine volume were relatively reduced, which has improved the patient’s health. This experiment has proved the excellent ability of goal directed fluid therapy combined with rehabilitation therapy in the treatment of esophageal cancer, and this research result has also proved the excellent medical effect of goal directed fluid therapy technology. Similarly, this paper also provided valuable reference information for the treatment of esophageal cancer.

## 1 Introduction

In the medical field, esophageal cancer is a common malignant tumor, which usually occurs on the inner wall of the esophagus. In recent years, great progress has been made in the treatment of esophageal cancer, creating opportunities for improving the survival rate of patients. However, there are still many difficulties in the treatment of esophageal cancer, such as the standardization of the scope of surgical resection and lymph node resection, and the early detection rate of esophageal mucosal and submucosal lesions. Therefore, the treatment of esophageal cancer needs to be further optimized and improved. The research direction of this topic is to use GDFT technology to improve the efficacy of cancer cell therapy. This study not only optimizes the use of medical fluids in esophageal cancer data, but also improves the repair effect of DNA damage, which has made a great contribution to the optimal treatment of cancer. Therefore, with the help of the current advanced GDFT technology, the treatment experience and effect of esophageal cancer can be greatly optimized.

Cancer is a common malignant tumor with high incidence and poor therapeutic effect. Nowadays, many scholars have conducted more scientific research on cancer. Strickler John H pointed out that the “liquid biopsy” method of cell-free DNA (cfDNA) in the blood of cancer patients was increasingly used in clinical practice. His research showed that the frequency of genomic changes detected in cfDNA was comparable to that observed in three independent tissue based colorectal cancer sequencing pharmacopoeias. His analysis also found a group of new extracellular domain mutations that mediated drug resistance by blocking the binding of anti cellular antibodies (Strickler et al., 2018). Cai et al. (2017)’s research pointed out that reversing abnormal DNA methylation and related gene silencing by inhibiting DNA methyltransferases (DNMTs) was an important potential cancer treatment method. He not only defined the leading role of DNA methyltransferase DNMT1, but also defined its different roles in the maintenance of genome wide DNA methylation. Irianto et al. (2017) found that the migration of micron scale narrowing broke the nucleus. By releasing the green fluorescent protein (GFP) located in the nucleus, he observed that for wild type and subclonal U2OS cells, the nuclear damage induced by migration was reversible. In addition to the persistent genomic differences between stable clones revealed by DNA array and sequencing, the acquisition and loss of hundreds of megabases in many chromosomes were typical changes and heterogeneity in bone cancer. Su et al. (2020) believed that accurate cancer cell recognition and effective treatment were very necessary and challenging in clinical practice. He reported the first example of DNA tetrahedral nanostructures (DTNs), real-time monitoring and imaging of three kinds of intracellular miRNAs based on fluorescence “off” to “on” mode, and cancer treatment induced by miRNA silencing. DTNs were self assembled from seven customized single stranded nucleic acid chains containing three target miRNAs recognition sequences. In addition, he studied the correlation between inhibiting migration and invasion of cancer cells and inducing apoptosis of cancer cells, and predicted that the development of intelligent nano platforms would open a door for cancer diagnosis and treatment. The above research topics have studied cancer research from multiple directions, which has provided great help for the research work of this paper. However, the contents of these research papers do not combine cancer research with GDFT technology. Therefore, further research is needed.

Nowadays, with the rapid development of science and technology, a variety of intelligent technologies have been applied to multiple cross cutting research topics. Therefore, GDFT technology can be applied to the treatment research, which provides more advanced research for cancer treatment. At present, many scholars have studied the correlation between liquid therapy technology and cancer. Yin et al. (2021) studied the correlation between GDFT and the treatment of adenocarcinoma and tumor. He evaluated somatic mutations, circulating tumor cells (CTC) and circulating tumor DNA (ctDNA) in patients with pancreatic ductal adenocarcinoma (PDAC) who had pathological complete response (pCR) after adjuvant therapy, and found that they were related to prognosis. In his study, he pointed out the existence of somatic mutation, CTC and ctDNA even in patients with pCR to PDAC, which might predict the early recurrence probability of cancer. Sivakumar et al. (2022) pointed out that GDFT technology and genome mapping had changed breast cancer care. His research showed that cancer usually evolved and evaded therapeutic intervention by acquiring genomic mutations. He examined tissue and fluid biopsy patients as part of routine clinical care to characterize the evolution of the tumor and to identify potential vulnerabilities in the event of recurrence. In general, his research had provided insights into treatment and selection driven tumor evolution, and identified a potential combination treatment scheme for advanced breast cancer. It can be seen from the above research briefs of various scholars that their research results are of great reference value and provide a good direction for this study. However, their research also has some shortcomings. It can be seen that most of their research on knowledge technology theory, which leads to the lack of practicality and authenticity of their research results.

Esophageal cancer is a relatively common malignant tumor, and its incidence rate and mortality increase with age. At present, the treatment of esophageal cancer is a field that has been widely concerned by the society, and its development prospects are good. In addition to surgical treatment, esophageal cancer also has chemotherapy, radiotherapy and other means, but these can not cure the tumor, just to prolong the life of patients, and reduce their pain, thus improving the quality of life. In addition, because the early symptoms of esophageal cancer are not obvious, many patients are not found until the disease develops to the late stage. Therefore, early screening is very important. It is suggested that physical examination should be carried out in time to make early diagnosis, early treatment and control of the disease, so as to avoid serious consequences in the late stage. The innovation of this paper is to apply GDFT technology to the treatment of esophageal cancer, which can quickly detect tumor cells, quickly locate and accurately kill them, thus improving the survival rate and treatment experience of patients.

## 2 Treatment of esophageal cancer

### 2.1 Concept and pathology of esophageal cancer

Esophageal cancer refers to the cancer of the esophagus, and refers to the cancerous changes in the tissues of the esophagus. Esophageal cancer is a fatal cancer. It occurs in the esophagus, which is a long tube from the throat to the stomach (Adjiri, 2017; Chang et al., 2019). Esophageal cancer can affect people’s swallowing of food and drink, leading to dysphagia, chest pain and vomiting. Symptoms of esophageal cancer may also include coughing, dry throat, hoarseness, and throat discomfort. The cause of esophageal cancer is not completely clear, but there are some factors that may increase the risk, including smoking, excessive drinking, long-term exposure to smoke or toxins, eating many pickled foods, suffering from Egyptian peduncle (an oral cancer), throat infection or asthma. The treatment of esophageal cancer depends on the extent of cancer spread and health status. Common treatment methods include surgery, chemotherapy, radiotherapy and immunotherapy. Early detection and treatment of esophageal cancer is very important to improve the survival rate. The pathology of esophageal cancer has the following categories. Esophageal cancer can be divided into three types: esophageal adenocarcinoma, esophageal sarcoma and esophageal neuroendocrine carcinoma.

#### 2.1.1 Esophageal adenocarcinoma

Esophageal adenocarcinoma is a common malignant cancer, which usually occurs in the middle and lower part of the esophagus (Sato et al., 2017; Song et al., 2019). It is a cancer caused by gland cells (cells with secretory function) in the esophagus. The early symptoms of esophageal adenocarcinoma are often not obvious. Common symptoms include: throat discomfort, dysphagia, anorexia, weight loss, cough, dry throat, hoarseness, etc. If these symptoms occur, patients must seek medical attention as soon as possible. In order to determine the condition, the doctor may suggest some examinations, including gastroscopy, CT scanning, PET (positron emission tomography) scanning, etc., which can help the doctor understand the condition and decide on the treatment plan. The treatment of esophageal adenocarcinoma depends on the severity of the disease. Common treatment methods include surgery, chemotherapy and radiotherapy. If the condition is mild, only surgery may be required. If the disease is serious, chemotherapy and radiotherapy may be combined. The purpose of the operation is to remove the tumor in order to cure it. Chemotherapy and radiotherapy are carried out after surgery, aiming to eliminate cancer cells left in the body to prevent recurrence. The prognosis of esophageal adenocarcinoma depends on the severity of the disease. Early detection and treatment can improve the survival rate. It is recommended that regular physical examination be carried out to detect and treat esophageal cancer in time.

During the treatment of esophageal adenocarcinoma, patients may need to receive long-term chemotherapy and radiotherapy, which may cause a greater burden on the body. In order to reduce the burden, patients may need to receive nutritional support treatment to maintain health. In addition, during treatment, the patient may have difficulty swallowing, which can lead to malnutrition and weight loss. To solve this problem, the doctor may suggest that patients use enteral nutrition or gastrointestinal infusion, or use swallowing aids. In general, the treatment of esophageal adenocarcinoma is a complex process, which requires comprehensive consideration of many factors. During the treatment, the patient needs to closely cooperate with the doctor and actively cooperate with the treatment. At the same time, they should also pay attention to their physical health and maintain a good psychological state to help them cope with the challenges posed by treatment (Savoli and Bhatt, 2022).

#### 2.1.2 Esophageal sarcoma

Esophageal carcinoma, also known as esophageal sarcoma, is a relatively common malignant tumor, which occurs in the inner wall of the esophagus (Short et al., 2017; Ma et al., 2019). The esophagus is the tube connecting the throat and stomach in the human body, and food is transported to the stomach through the esophagus during eating. When esophageal cancer occurs, the tumor grows in the inner wall of the esophagus and may spread to surrounding tissues and organs. There are many symptoms of esophageal cancer. Common symptoms include sore throat, food blockage, cough, vomiting, anorexia, and significant weight loss. If these symptoms are found, patients should go to the hospital immediately for diagnosis and treatment. There are many ways to treat esophageal cancer, including surgery, chemotherapy, radiotherapy, and immunotherapy. The specific treatment plan depends on the specific situation of the patient and the severity of the tumor. If found and treated in time, the cure rate of esophageal cancer is high. Therefore, if the above symptoms are found, patients should go to the hospital immediately for diagnosis and treatment. In addition to surgery, chemotherapy, radiotherapy and immunotherapy, nutritional support for patients with esophageal cancer is also very important. Since patients with esophageal cancer may suffer from anorexia and significant weight loss, nutritional support should be paid attention to. Nutritional support includes oral nutrition liquid, enteral nutrition liquid, parenteral nutrition liquid, etc. These nutrient solutions are composed of various nutrients, which can help patients supplement nutrition and maintain health.

In addition, for patients with esophageal cancer, smoking and drinking should be avoided as far as possible. Smoking and drinking are both risk factors of esophageal cancer, which can increase the risk of esophageal cancer. Therefore, patients should avoid smoking and drinking as much as possible to reduce the risk of esophageal cancer. In a word, esophageal cancer is a serious disease. If it is found and treated in time, the cure rate is high. Therefore, if there are relevant symptoms, patients should go to the hospital immediately for diagnosis and treatment.

#### 2.1.3 Esophageal neuroendocrine carcinoma

Esophageal neuroendocrine carcinoma is a rare cancer, which occurs in the neuroendocrine cells in the esophagus (Sohda and Kuwano, 2017; Yang et al., 2020). These cells are secreted by hormones, which can control and regulate digestion, endocrine and physiological functions. Esophageal neuroendocrine carcinoma is rare, but when it occurs, it can cause serious problems. The incidence rate of this cancer is very low, but if not treated in time, it may lead to death. Symptoms of esophageal neuroendocrine carcinoma may include chest pain, dyspnea, sore throat, pharyngeal discomfort, dysphagia, neck mass, weight loss, and weight loss. The diagnosis of this cancer is usually made by chest X-ray, computed tomography, magnetic resonance imaging and radionuclide scanning. The treatment of esophageal neuroendocrine carcinoma may include surgery, chemotherapy, radiotherapy and targeted therapy. The choice of these treatments depends on the location, size and expansion of the cancer and the health of the patient. If esophageal neuroendocrine carcinoma is diagnosed and treated early, the survival rate of patients may be higher. Therefore, if one has the above symptoms, please consult doctors for help as soon as possible.

During the treatment of esophageal neuroendocrine carcinoma, patients may need to receive supportive treatment to help them cope with the side effects of treatment and improve their quality of life. These supportive treatments may include nutritional support, pain management, psychological support and lifestyle intervention. It is an important part of the treatment of esophageal neuroendocrine carcinoma to keep close communication with doctors. Patients should discuss treatment options and expected results with doctors, and report any problems or concerns during treatment. In general, esophageal neuroendocrine carcinoma is a rare but serious disease. Early diagnosis and treatment can improve the survival rate and prognosis of patients. It is recommended to consult doctors for more information and treatment options on esophageal neuroendocrine carcinoma.

**FIGURE 1:**
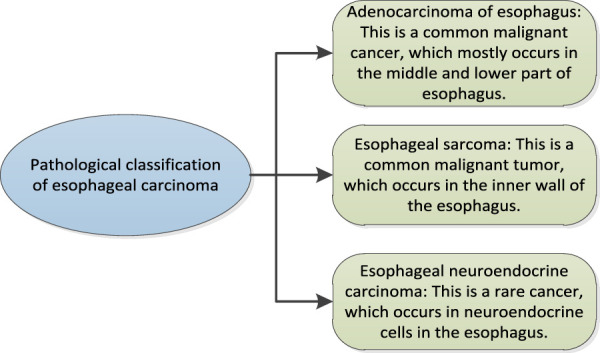
Pathological classification of esophageal cancer.

The pathological classification of esophageal cancer is shown in Figure 1.

### 2.2 DNA damage

DNA damage refers to the damage to DNA molecules. DNA molecule is the genetic material of cells, responsible for storing and transmitting genetic information. When DNA is damaged, it may lead to the loss or change of genetic information, leading to cell function defects or cell canceration. DNA damage may be caused by many different factors, including chemical damage, physical damage and biological damage. Chemical damage may come from natural or man-made chemical substances. Physical damage may come from radiation or high temperatures. Biological damage may result from bacterial or viral infection. If DNA damage cannot be repaired, the following consequences may result.

#### 2.2.1 Physiological function disorder

Cell proliferation refers to the process of cell division and is the basis of cell growth and organism growth. When cell proliferation decreases, it may lead to genomic instability. Genome refers to the combination of all genes of an organism, which endows the organism with characteristics, morphology and functions. When the genome is unstable, it may lead to changes in genes in cells, which may lead to physiological dysfunction. These physiological disorders may include physical development disorder, immune function disorder, metabolic function disorder, etc. In addition, when cell proliferation decreases, leading to genomic instability and physiological dysfunction, many different symptoms may occur. These symptoms may include weight loss, fatigue, anemia, skin itching, immune dysfunction, etc. If treatment cannot be carried out in time, this situation may lead to more serious consequences, even life threatening (Zhao et al., 2021). In order to avoid this, it is recommended to maintain a healthy lifestyle, including a balanced diet, proper physical exercise and avoiding harmful factors in the natural environment, such as radiation and pollution. If people find any abnormal symptoms, they should go to a doctor as soon as possible so that they can get treatment in time.

#### 2.2.2 Cell death

DNA is a kind of information carrier in organism, which stores the genetic information of organism. DNA damage is very common in cells, which can be caused by many factors, including radiation, toxins and other environmental pressures. When DNA is damaged, it may not be able to perform its function normally, which may lead to abnormal signal transmission. Signal transduction is a kind of communication mechanism in organism, which enables information in cells to be transmitted to cells through signal transduction, so as to coordinate cell activities. When DNA is damaged, it may lead to abnormal signal transmission, which means that information in cells cannot be transmitted normally. This abnormal signal transduction may lead to cell death. Cell death refers to the process in which cells stop living and gradually decompose. Generally speaking, cell death is a normal physiological process. However, if it occurs excessively, it may lead to tissue damage or disease. In the case of abnormal signal transduction caused by DNA damage, cells may die because they cannot receive the necessary signals. This cell death may lead to a decline in tissue function and may, in some cases, lead to disease. On the other hand, if the DNA damage response pathway is overactive or dysfunctional, it may also lead to cell death or other negative results. In general, signal pathways involved in DNA damage response play a crucial role in maintaining genome integrity and ensuring cell survival. The maladjustment of these pathways has serious consequences for the health of organisms.

#### 2.2.3 Abnormal cell proliferation and metabolism

DNA is a macromolecule, which stores the blueprint of biological genetic information. This information is contained in DNA sequences and guides protein synthesis through transcription and transformation. Cells usually have the ability to repair DNA damage. However, if the DNA damage is too much or the repair mechanism is damaged, this may lead to abnormal gene expression, and then lead to abnormal cell proliferation and metabolic abnormalities. When DNA is damaged, these information may be changed or destroyed, which may lead to abnormal gene expression. Abnormal gene expression refers to changes in the level of gene activity, which may lead to abnormal cell proliferation and metabolic abnormalities. For example, if the expression level of a gene is too high or too low, it may lead to abnormal cell proliferation, leading to tumor formation. Similarly, if abnormal gene expression leads to changes in the activity of some enzymes, this may lead to abnormal metabolism and have a negative impact on the health of organisms.

In general, DNA damage has a huge impact on human health. As the genetic material of cells, DNA stores the genetic information of all cells and guides how cells divide, grow and function. Finally, in the long run, when DNA is damaged, cells may not function normally, which may lead to a series of problems, including aging, tumors and diseases.

**FIGURE 2:**
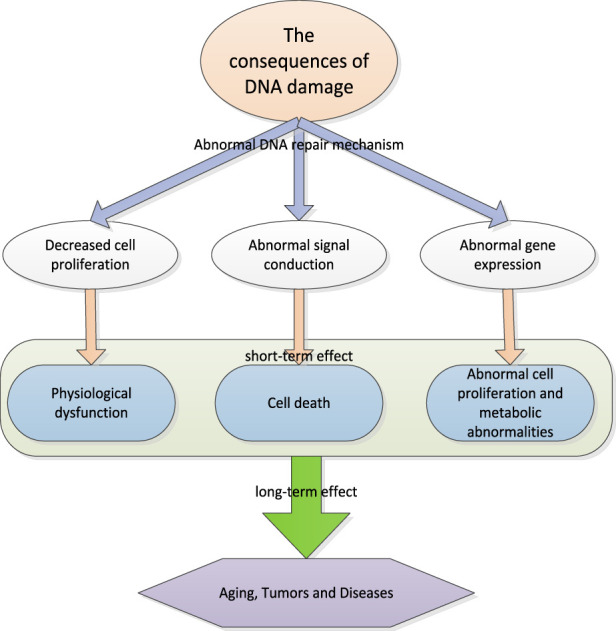
Consequences of DNA damage.

The consequences of DNA damage are shown in Figure 2.

### 2.3 Cancer treatment and DNA damage repair

Cancer treatment refers to the treatment of patients with cancer, aiming to inhibit or eliminate the growth and reproduction of cancer cells (Kopinski et al., 2021). Cancer is a disease caused by DNA damage. Therefore, one of the ways to cure cancer is to eliminate cancer cells by repairing DNA damage. DNA damage repair means that after DNA damage occurs, the repair mechanism in the cell can repair and restore DNA to its original state. This repair process can be implemented in many ways, such as direct repair, alternative repair, and deletion after repair. These repair mechanisms are completed by special repair enzymes, such as multifunctional repair enzymes and transferases. DNA damage repair is also widely used in cancer treatment. For example, chemotherapy drugs damage DNA. Therefore, in the process of chemotherapy, the DNA damage repair mechanism in cells plays an important role to protect cells from damage. In addition, some drugs can also directly act on the DNA damage repair mechanism to make it more effective. DNA damage repair is an important biological process, which is carried out in cells to repair DNA damage, so as to protect the integrity of the genome and the normal function of cells. In the treatment of cancer, DNA damage repair can start from the following aspects.

#### 2.3.1 Blocking repair pathway

Blocking of repair pathway refers to the process of blocking DNA repair pathway with drugs or other methods. This method allows drugs to kill cancer cells more effectively, because cancer cells usually have higher DNA repair capacity than normal cells. For example, PARP (Poly ADP-ribose Polymerase) inhibitors can block the activity of PARP repair pathway, which is a method commonly used to treat breast cancer and ovarian cancer. PARP is a protein that plays an important role in DNA repair process and is usually used by cancer cells to repair DNA damage. Therefore, the blocking of PARP repair pathway can make drugs more effective in killing cancer cells. Another commonly used method to block the repair pathway is to use TOPO II inhibitors, which is a method used to treat leukemia and tumor. TOPO II is a protein that plays an important role in DNA repair. Therefore, the blocking of TOPO II repair pathway can make the drug more effective in killing cancer cells. In general, the blocking of the repair pathway is an effective way to treat cancer, which can make drugs more effective in killing cancer cells, but may also lead to side effects, so it needs to be used with caution (Arslan and Koyuncu, 2021).

#### 2.3.2 Activating repair path

The activation of repair pathway refers to the process of activating DNA repair pathway by drugs or other methods. This method can help normal cells better repair DNA damage, thereby reducing the risk of cancer. For example, ATM (Ataxia telangiectasia mutated) activator can activate ATM repair pathway, which is a method commonly used to prevent cancer and promote DNA repair. ATM is a protein that plays an important role in DNA repair and is usually used by normal cells to repair DNA damage. Therefore, the activation of ATM repair pathway can help normal cells better repair DNA damage, thereby reducing the risk of cancer. Another common way to activate the repair pathway is to use DNA repair enzymes to directly repair DNA damage, which in some cases can help reduce the risk of cancer. In general, activation of the repair pathway is an effective way to prevent cancer, which can help normal cells better repair DNA damage. However, there are not enough studies to prove its effectiveness in the treatment of cancer.

#### 2.3.3 Taking advantage of weaknesses in the repair approach

The weakness of the repair approach refers to the method of treating cancer by using the loopholes or defects of the repair approach. This method is based on the fact that cancer cells usually have higher DNA repair capacity than normal cells. Therefore, in some cases, the use of weaknesses in the repair pathway can be exploited to make drugs more effective in killing cancer cells. For example, BRCA (breakthrough cancer susceptibility gene) mutation leads to defects in BRCA repair pathway. Therefore, BRCA mutated cancers may be sensitive to PARP inhibitors. PARP inhibitors are drugs that can block the repair pathway of PARP. Therefore, the use of the weakness of BRCA repair pathway can enable PARP inhibitors to kill cancer cells more effectively. Another common way to exploit the weaknesses of the repair pathway is to use DNA repair enzyme mutants. These enzyme mutants have higher efficiency than normal enzyme mutants in repairing DNA damage, so they can be used to treat certain types of cancer. In general, the use of the vulnerability of the repair pathway is an effective way to treat cancer, but further research is needed to determine its effect in different situations. In general, there is an inseparable relationship between cancer treatment and DNA damage repair, and the effectiveness of DNA damage repair is one of the important factors determining the effect of cancer treatment. Through in-depth study of the mechanism of DNA damage repair, more effective drugs and therapies can be developed to improve the efficiency of cancer treatment. In addition, DNA damage repair may also be related to some other diseases, such as hereditary diseases, senile diseases, etc. Therefore, the research on DNA damage repair is not only helpful to improve the efficiency of cancer treatment, but also may provide new therapeutic methods and ideas.

**FIGURE 3:**
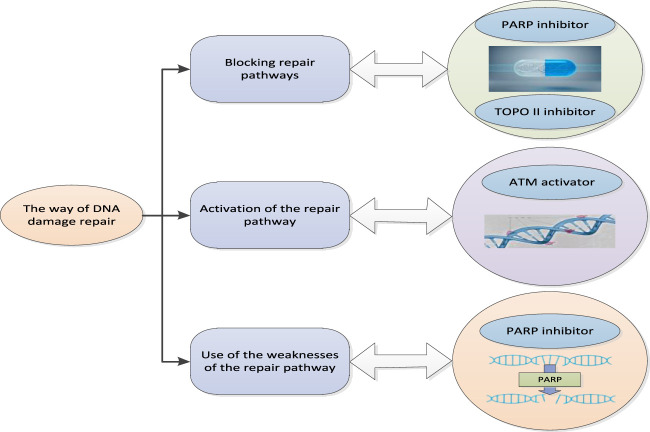
Pattern of DNA damage repair.

The DNA damage repair method is shown in Figure 3.

## 3 GDFT combined with rehabilitation therapy technology

### 3.1 Target oriented liquid therapy

Targeted liquid therapy is a medical technology, which aims to use liquid or gas to directly inject drugs or other substances into specific target areas of the body to treat certain diseases or restore body functions. This technique is often used to treat diseases in organs or tissues, such as tumors, inflammation or infections.

In target oriented liquid therapy, doctors use imaging technology (such as ultrasound, magnetic resonance imaging or computed tomography) to help determine the location of the treatment target area. Then, the doctor makes a small incision in the skin and uses a guide to inject drugs or other substances into the target area. In some cases, it may be necessary to use multiple imaging techniques to help doctors locate the target area more accurately. Other equipment, such as a liver separator or liver cancer treatment device, may also be needed to help inject drugs into the target area. Targeted liquid therapy may be used to treat various diseases, including tumor, inflammation, and infection. It may also be used to restore body functions, such as improving liver function through injections of drugs or promoting blood circulation through injections. The advantages of goal oriented liquid therapy include accuracy and pertinence. Because the drug is directly injected into the target area, it can reduce the impact on other parts and reduce the risk of side effects. However, this technology also has some limitations. For example, it may not be suitable for some diseases, or it needs to use special equipment for treatment.

In the goal oriented liquid therapy, doctors use certain liquid drugs, such as biological agents with precise formula and use, to directly treat specific diseases of patients. This method can reduce the damage to other healthy tissues and make the drug more effective. In addition, goal oriented liquid therapy mainly guides liquid therapy through “fluid response.” Liquid reaction refers to the chemical reaction between drugs and cells, tissues or organs in the human body. These reactions may lead to changes in the mechanism of action, bioavailability or toxicity of the drug. For example, when a drug enters the human body, it may react with some enzymes or proteins to change the mechanism of action of the drug. These liquid reactions can be studied through chemical analysis, biological experiments or pharmacological experiments. In addition, the fluid response is to observe the changes of cardiac output and other indicators after a certain volume of fluid is given to the body. If cardiac output increases, fluid infusion continues, otherwise, it stops.

**FIGURE 4:**
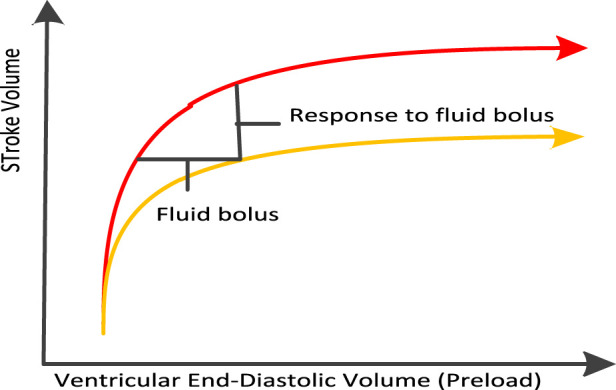
Frank starling curve of liquid reaction.

The Frank Starling curve of liquid reaction is shown in Figure 4.

### 3.2 EEG signal optimization algorithm based on combined rehabilitation therapy

EEG signal is the physiological signal of human brain, which can reflect human cognitive activity, emotional state, memory, attention level, and other information. The optimization algorithm of EEG signal usually refers to the method of processing, analyzing and visualizing EEG signal to improve signal quality, and enhance information content and signal interpretability.

In order to ensure the accuracy of EEG signals, it is necessary to denoise EEG signals. Since the signal has been processed by power frequency denoising, low-pass filtering and baseline removal, this section directly removes the adaptive eye artifacts.

First, three channel signals ( $o_{1}$ , $o_{2}$ , and $o_{3}$ ) are used for image artifact removal. The calculation formula is as follows:

$$a\left(t\right)=j\left(t\right)+\left\{o_{1}\left(t\right),o_{2}\left(t\right),o_{3}\left(t\right)\right\}b$$ (1)

Among them, $a\left(t\right)$ represents a certain EEG channel signal. $j\left(t\right)$ represents the filtered value of this channel. b represents the weight of EEG artifacts on this channel. The filtering model of multi-channel EEG signal can be expressed as:

$$A_{T*M}=J_{T*M}+O_{T*N}*B$$ (2)

Among them, EEG signal A and filtered EEG signal J have M channels respectively, and the length of each channel is T. The ophthalmic noise O has N channels, and B represents the channel weight.

Therefore, the filtered target signal can be expressed as:

J=A−OB (3)

At the same time, the left and right sides calculate the covariance of the noise matrix to obtain the formula:

$$Cov\left(O,J\right)=Cov\left(O,\left(A-OB\right)\right)$$ (4)

Since signal J and noise O are independent, their cross covariance is zero. Therefore, the weight B can be expressed as:

$$B=D_{NN}*D_{NY}$$ (5)

Among them, $D_{NN}$ is the autocovariance matrix of EEG signal, and $D_{NY}$ is the cross covariance matrix of EEG signal.

Through the above algorithm formulas, EEG signals can be optimized. EEG signal optimization algorithm is a technology used to improve the quality of EEG signal. It improves the accuracy and clarity of EEG signals by reducing the influence of interference signals, improving the signal to noise ratio, speeding up signal processing, improving the reliability and stability of signals, and reducing the noise of EEG signals. These optimization measures can better help researchers analyze and understand EEG signals, so as to help better study brain functions and diseases.

## 4 Implementation and testing of esophageal cancer treatment based on GDFT combined with rehabilitation therapy technology

### 4.1 Testing of EEG signal optimization algorithm based on combined rehabilitation therapy

After the EEG signal optimization algorithm based on combined rehabilitation therapy is proposed, it can not only rely on theory, but also need to test the calculation effect of the algorithm in practical use. This experiment tests the traditional EEG signal algorithm and the EEG signal optimization algorithm based on the combined rehabilitation treatment.

First, the measurement accuracy of these two algorithms for common EEG waves need to be counted: Delta, Theta, Alpha, Beta and Gamma. The test results are shown in Table 1.

**TABLE 1:** Measurement accuracy of EEG.

	Traditional brain-wave signal algorithm (%)	Optimization algorithm of brainwave signal based on combined rehabilitation therapy (%)	Differential value (%)
Delta	88.6	94.8	6.2
Theta	89.4	94.1	4.7
Alpha	90.8	95.2	4.4
Beta	88.2	94.8	6.6
Gamma	90.2	95.2	5.0

It can be seen from the test results in Table 1 that the measurement accuracy of EEG signal optimization algorithm based on combined rehabilitation treatment for various EEG signals is higher than that of traditional EEG signal algorithm. Among them, the biggest difference in measurement accuracy is the beta EEG measurement. The measurement accuracy of beta EEG by traditional EEG signal algorithm and EEG signal optimization algorithm based on combined rehabilitation treatment is 88.2% and 94.8% respectively, with a difference of 6.6%.

**FIGURE 5:**
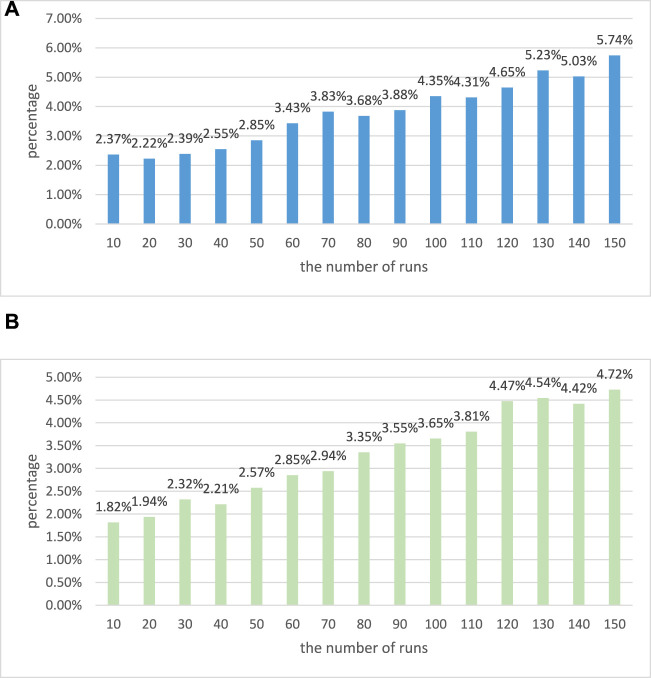
Operation error diagram of two algorithms. **(A)** Operation error diagram of traditional EEG signal algorithm. **(B)** Operation error diagram of EEG signal optimization algorithm based on combined rehabilitation therapy.

In addition, it is also necessary to investigate the measurement error rate of these two algorithms in a long running environment. The results are shown in Figure 5.

It can be seen that the measurement error rates of these two algorithms are increasing when running for a long time. However, it can be seen that the measurement error rate of the traditional EEG signal algorithm in each run is greater than that of the EEG signal optimization algorithm based on combined rehabilitation therapy. When the number of runs is 150, the measurement error rates of these two algorithms are 5.74% and 4.72% respectively. Through these tests, it can be shown that the EEG signal optimization algorithm based on combined rehabilitation therapy has better performance.

### 4.2 Experiment based on GDFT combined with rehabilitation technology in the treatment of esophageal cancer

#### 4.2.1 Experimental direction

The research direction selected in this paper is to test the effect of GDFT combined with rehabilitation technology in the treatment of esophageal cancer, and its therapeutic effect on esophageal cancer patients is explored.

#### 4.2.2 Experiment content

Adults who are selected for esophageal cancer surgery are randomly divided into two groups. During the operation, the patients are treated with GDFT and conventional therapy, and their physical conditions are observed.

#### 4.2.3 Experimental methods

In this survey, the comparative survey mode is adopted to ensure the scientific and effective experiment.

#### 4.2.4 Experimental results

First, two groups of patients with esophageal cancer are selected and their basic information is collected for statistics, as shown in Table 2.

**TABLE 2:** Comparison of basic information of two groups of patients before treatment.

	GDFT treatment group	Conventional treatment group
Gender ratio (male/female)	52.8%	53.2%
Average age(year)	52.7	52.5
Average BMI(body mass index) (kg/m^2^)	22.32	22.66
Average BIS(Bispectral index)	97.56	97.84

From the basic information of esophageal cancer in Table 2, it can be seen that the gender ratio, average age, average body mass index and average EEG bispectral index of the GDFT treatment group and the conventional treatment group are very close. This shows that the basic information gap between the two groups of patients with esophageal cancer is small, ensuring the authenticity of the follow-up experiment.

**FIGURE 6:**
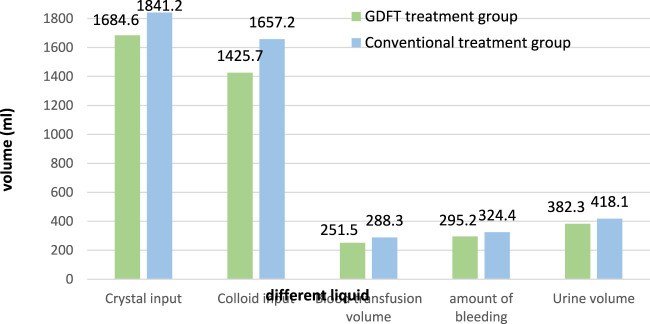
Fluid input and output of two groups of patients during treatment.

During the investigation, first of all, the liquid intake and output of these two treatment technologies during treatment are investigated. The results are shown in Figure 6.

It can be seen from Figure 6 that in the treatment of esophageal cancer patients, the crystal input volume of GDFT treatment and conventional treatment is 1,684.6 mL and 1841.2 mL respectively, and their colloid input volume is 1,425.7 mL and 1,657.2 mL respectively. In addition, their blood transfusions are 251.5 mL and 288.3 mL, respectively. This shows that after GDFT treatment, the input amount of various medical fluids is relatively reduced, and the use of medical fluids is more economical. In addition, there is a comparison of liquid output. Their bleeding volume is 295.2 mL and 324.4 mL respectively, and their urine volume is 382.3 mL and 418.1 mL respectively. This shows that after GDFT treatment, the patient’s blood loss and urine volume are relatively reduced, which improves the patient’s health.

**FIGURE 7:**
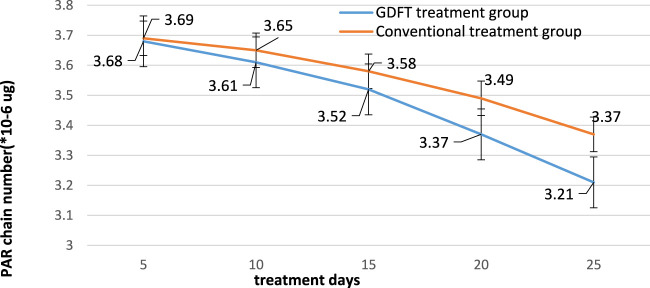
DNA damage repair of two groups of patients.

After that, the DNA damage repair of the patient needs to be investigated after using the two treatment schemes. poly adp-ribose polymerase (PARP) can detect single strand breaks in DNA and initiate DNA damage repair reaction. Once single strand breaks are detected, PARP combines with DNA and starts to generate PAR chains as signals for other DNA repair enzymes. The test results are shown in Figure 7.

According to the data in Figure 7, it can be seen that the number of PAR chains in these two groups of patients decreases with the increase of treatment days after the use of GDFT treatment and conventional treatment. Their PAR chains have decreased from 3.68 * 10-6ug and 3.69 * 10-6ug to 3.21 * 10-6ug and 3.37 * 10-6ug, respectively. This shows that these two groups of treatment technologies can effectively treat patients’ DNA damage. In addition, it can be seen from the figure that the number of PAR chains in the GDFT treatment group is significantly reduced, which proves that GDFT treatment is more effective in repairing DNA damage.

## 5 Conclusion

Combined rehabilitation therapy is a comprehensive rehabilitation treatment method that integrates behavior correction, drug therapy and physical therapy to help patients overcome bad behavior habits and improve the quality of life. The combined rehabilitation treatment includes several aspects: exercise, sleep, stress management, diet management and emotion management, which help to improve the quality of life, and eliminate psychological stress to improve social support, so that patients can better cope with the challenges brought by the disease, and eventually recover to a good state of life. The rehabilitation therapy based on GDFT has a very excellent effect in the treatment of esophageal cancer, and its experimental effect is very significant in the real medical environment. In addition, the EEG signal optimization algorithm proposed in this paper based on the combined rehabilitation treatment also had a good effect. It has been proved through experiments that after using the EEG signal optimization algorithm based on combined rehabilitation therapy, the accuracy of various EEG measurements has been effectively improved. According to a variety of experimental results, the combination of GDFT and rehabilitation technology had excellent medical effect in the treatment of esophageal cancer. After using the combined rehabilitation technology based on GDFT, the fluid intake and output of the patients were optimized. In addition, it also had excellent repair effect on DNA damage. In conclusion, this paper clearly discussed the good therapeutic effect of GDFT combined with rehabilitation therapy in esophageal cancer, which has provided a favorable research direction for cancer treatment.

## Data Availability

The original contributions presented in the study are included in the article/supplementary material, further inquiries can be directed to the corresponding author.
